# Age-associated alterations in inducible gene transcription in human CD4^+^ T lymphocytes

**DOI:** 10.18632/aging.100522

**Published:** 2013-01-30

**Authors:** Arsun Bektas, Yongqing Zhang, William H. Wood, Kevin G. Becker, Karen Madara, Luigi Ferrucci, Ranjan Sen

**Affiliations:** ^1^ Laboratory of Molecular Biology and Immunology, National Institute on Aging, National Institutes of Health, Baltimore, MD 21224, USA; ^2^ Translational Gerontology Branch, National Institute on Aging, National Institutes of Health, Baltimore, MD 21224, USA; ^3^ Research Resources Branch, National Institute on Aging, National Institutes of Health, Baltimore, MD 21224, USA; ^4^ Clinical Research Branch, National Institute on Aging, National Institutes of Health, Baltimore, MD 21224, USA

**Keywords:** human, CD4^+^ T cell, NF-κB, aging, aene expression

## Abstract

Age associated immune dysregulation results in a pro-inflammatory state and increased susceptibility to infections and autoimmune diseases. Studies show that signaling initiated at the T cell antigen receptor (TCR) is impaired in CD4^+^ T cells from old compared to young mice. Here we examined TCR-inducible gene expression changes in CD4^+^ T cells during human aging. We reveal a dichotomy in gene expression mediated by the inducible transcription factor NF-κB. Most NF-κB target genes are not induced in a sustained manner in cells derived from older compared to younger individuals. However, a subset of NF-κB target genes including genes associated with chronic pro-inflammatory state in the elderly, such as interleukin 1 and 6, continue to be up-regulated even in the absence of NF-κB induction. In addition, we identify other widespread changes in gene expression between cells derived from older and younger individuals. Surprisingly, many of the most noteworthy age-associated changes in human CD4^+^ T cells differ from those seen in murine models. Our studies provide the first view of age-associated alteration of TCR-inducible gene expression in human CD4^+^ T cells.

## INTRODUCTION

Human aging is associated with chronic low-grade inflammation marked by increased levels of pro-inflammatory cytokines IL-1, IL-6 and TNFα [1-3]. Elevated levels of these inflammatory markers are powerful risk factors for accelerated decline of physical and cognitive function, and mortality. Because genes encoding these cytokines are targets of the transcription factor NF-κB, it has been suggested that dysregulation of this factor is a central feature of the aging process [4, 5]. The most prominent role of NF-κB is as an inducible transcription factor [6, 7]. In the immune system, NF-κB is activated in response to antigen receptor signals, to Toll-like receptor signals and to a variety of cytokines. Additionally, reactive oxygen species (ROS) are potent activators of NF-κB in diverse cell type [8, 9]. The connection to ROS provides another possible link between NF-κB and aging [10]. Biochemical evidence for alteration of NF-κB during aging was first obtained in rodents where NF-κB DNA binding was shown to be elevated in tissues from older animals [11-14]. More recently, elevated basal NF-κB has also been detected in primary human dendritic cells, endothelial cells, and in skeletal muscle [15-17]. However, the effect of increased basal NF-κB on gene expression has not been examined in most cases.

Surprisingly few studies have coordinately examined the effect of age on NF-κB induction and NF-κB-dependent inducible gene expression. In their pioneering studies, Ponnapan and colleagues showed that TNFα-induced NF-κB DNA binding activity was impaired in T lymphocytes isolated from the elderly [18, 19]. However, NF-κB-dependent changes in gene expression were not examined in these studies. More recently, Huang et al. found that CD4^+^ T cells from older mice produced higher levels of pro-inflammatory IL-17 and IL-6 in response to TCR signaling [20]. This effect was reduced by anti-oxidants and pharmacological inhibitors of canonical NF-κB activation, leading to the proposal that NF-κB responses were increased with age. The contrasting observations in these papers could be due to differences in the activating stimuli (TNFα versus anti-CD3 and anti-CD28), differences between murine and human cells, or differences in responses between CD4^+^ and total T cells. Because immune impairment remains a key health issue in the elderly, it is imperative to clarify molecular mechanisms that contribute to age-associated reduction in immune responses.

Several earlier studies, mostly with murine lymphocytes, have shown that TCR signaling is defective in T cells obtained from older mice [21, 22]. These defects include reduced synapse formation and TCR recruitment to plasma membrane rafts, and reduced activation of cytoplasmic MAP kinase signaling. However, few studies have connected the consequences of age-associated TCR signaling defects to changes in inducible gene expression. Han et al. studied the TCR responsiveness of total CD3^+^ T cells from young and old mice [23]. They found that IFNγ was induced to significantly higher levels in cells from old mice, whereas MIP-1α and MIP-1β (CCL3 and CCL4, respectively) induction was somewhat higher in the older cells. Analysis of the CD4^+^ compartment by Yung and colleagues showed that certain chemokines (such as RANTES, MIP-1α and MIP-1β) and chemokine receptors (such as CCR2, CCR5 and CXCR5) were expressed at higher levels in unstimulated cells from old mice [24, 25]. However, long-term stimulation via the TCR showed comparable changes in cells from young or old mice in these studies. Recently, Mirza et al. carried out a comprehensive analysis of inducible gene expression in naïve murine CD4^+^ T cells obtained from young and old mice [26]. Genes and pathways that were affected with age included those associated with cell cycle, cell death, and inflammation. Chemokine genes that were up-regulated with age included *CCL3* and *4*, *CCL1* and *17* and *CXCL9* and *11*. Cytokine genes that were up-regulated with age included *IL-4* and *IL-10* and *IFN-γ*. Despite the variations, these studies demonstrate that TCR signaling defects translate into gene expression defects in T lymphocytes from old mice. Corresponding studies of human aging are lacking. Here we use biochemical assays and genome-wide RNA profiling to provide the first analysis of age-associated alterations in TCR-inducible gene expression in human CD4^+^ cells. Subjects for these studies were selected from the Baltimore Longitudinal Studies on Aging (BLSA), which has strict health criteria for enrollment eligibility. Additionally, cells were obtained from individuals not included in the BLSA but selected according to the same strict criteria for healthy status. Because of the exceptional health status of this population, changes seen in these populations can be confidentially attributed to aging *per se*.

Time-dependent response to T cell receptor stimulation showed that NF-κB induction was not sustained in CD4^+^ T cells from older donors. Gene expression analysis revealed a dichotomy in the regulation of NF-κB target genes. Activation of most target genes was not sustained in the older cohort, thereby closely recapitulating the biochemical studies. However, a subset of target genes, including *IL-6*, were hyper-activated in cells from older donors. In addition, activation or repression of numerous other genes was affected in the older cohorts. These observations define the functional consequences of dysregulated TCR signaling that accompany human aging and may, in part, explain the simultaneous occurrence of low grade chronic inflammation coupled with reduced immune responses in the elderly.

## RESULTS

**Figure 1:**
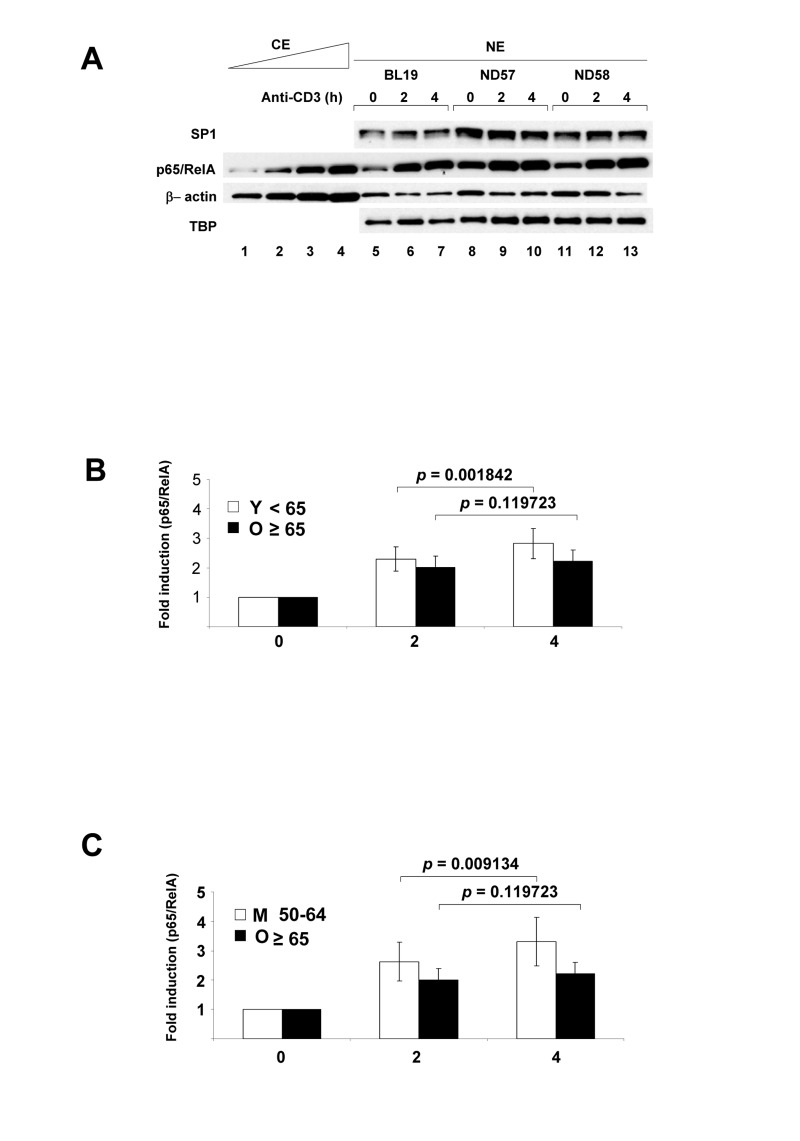
NF-κB p65/RelA nuclear expression in human peripheral blood CD4^+^ T lymphocytes activated via the T cell antigen receptor Purified CD4^+^ T cells were incubated at 37°C with plate-bound anti-CD3 for 2h or 4h; control cells were incubated for 4h without additional treatment. (**A**) Nuclear extracts (NE) were fractionated by SDS-PAGE, transferred to PVDF membranes and probed with antibodies directed against p65/RelA, transcription factors SP1 and TATA binding protein (TBP), or β actin. p65/RelA levels from 3 donors were compared to varying concentrations of a control cytoplasmic extract (CE). Induced p65/RelA levels in all 29 donors are shown in Table S3. (**B**) Fold-increase of nuclear p65/RelA treated cells was calculated relative to the levels in untreated cells (Table S4). The average fold increase of nuclear p65/RelA for 29 donors after 2h or 4h activation separated by subjects less than 65 years (Y) and 65 and over (O) is shown in the graph. *p*-values shown above bar graphs compare the fold induction between 2h and 4h of activation in Y and O groups, and were determined by Student's test (*p*<0.05 was considered as significant). Error bars reflect the standard error of the mean (±SEM) (in young 0.41; 0.52) (in old 0.38; 0.39). (**C**) Fold-increase of nuclear p65/RelA treated cells was calculated relative to the levels in untreated cells (Table S4). The average fold increase of nuclear p65/RelA for 21 donors after 2h or 4h activation separated by subjects 50-64 years (M) and 65 and over (O) is shown in the graph. *p*-values shown above bar graphs compare the fold induction between 2h and 4h of activation in M and O groups, and were determined by Student's test (*p*<0.05 was considered as significant). Error bars reflect the standard error of the mean (±SEM) (in middle 0.66; 0.83) (in old 0.38; 0.39).

We purified CD4^+^ T cells from 31 donors of age range 25-81 years (see Figure S1 and Table S1), and activated them with plate-bound anti-CD3 antibodies for 2h and 4h prior to preparation of nuclear and cytoplasmic extracts, and RNA. As controls, we used cells incubated for 4h at 37° in the absence of anti-CD3. We used anti-CD3 as an activating stimulus rather than IL-1 or TNFα in order to gain insight about age-associated attenuation of antigen-specific immune responses; the times of activation were chosen to limit the analysis to primary effects of TCR signaling. We first analyzed the response of the transcription factor NF-κB to TCR activation. Nuclear extracts were fractionated by SDS-PAGE, transferred to PVDF membranes and assayed for p65/RelA induction by immunoblotting (Figure 1A). To normalize between extracts we examined several ubiquitous proteins such as SP1, β-actin, TBP. We found that all of these were suitable for comparing extracts from one individual, but varied too much to compare between individuals (Figures S2A, S2B). To get an absolute measure of nuclear p65/RelA expression, we used a standard set of p65/RelA-expressing extracts (Figure S2C).

We compared nuclear p65/RelA induction in cells obtained from donors <65 years (Y) to ≥65 years (O). In the younger cohort, TCR activation resulted in a time-dependent increase of nuclear p65/RelA in both the 0-2 hour and 2-4 hour intervals (Figure 1B, Table S2, S3, S4). However, in the older group nuclear p65/RelA levels were not significantly different between 2h and 4h of activation. The same trend was also noted when total nuclear p65/RelA levels were compared between these age groups (Figure S3A). Because Y and O cells induced nuclear p65/RelA equally at 2h but not at 4h, we conclude that NF-κB activation in response to TCR stimulation is not effectively sustained in older individuals. During this activation time-course we did not observe nuclear induction of Rel.

It is well-recognized that CD4^+^ T cell subsets in peripheral blood change with age [27]. Notably, the proportion of naïve CD4^+^ T cells (expressing CD45RA antigen) decrease and the proportion of memory CD4^+^ T (expressing CD4RO antigen) increase with age. These changes stabilize within the third decade of life [28]. Accordingly, we did not observe significant differences in the RA/RO ratio in our cohort (Figure S1E). However, to circumvent possible effects of changing CD4^+^ T cell subsets we also compared NF-κB responsiveness in the ≥65 cohort with cells obtained from 50-64 year old donors. We found that NF-κB induction increased between 2h and 4h of activation even in this middle-aged group (M) (Figures 1C, S3B). We conclude that the observed difference in NF-κB responsiveness between the 50-64 age group and the ≥65 group reflects a true reduction in the response of the CD4^+^ cells obtained from older donors.

**Figure 2:**
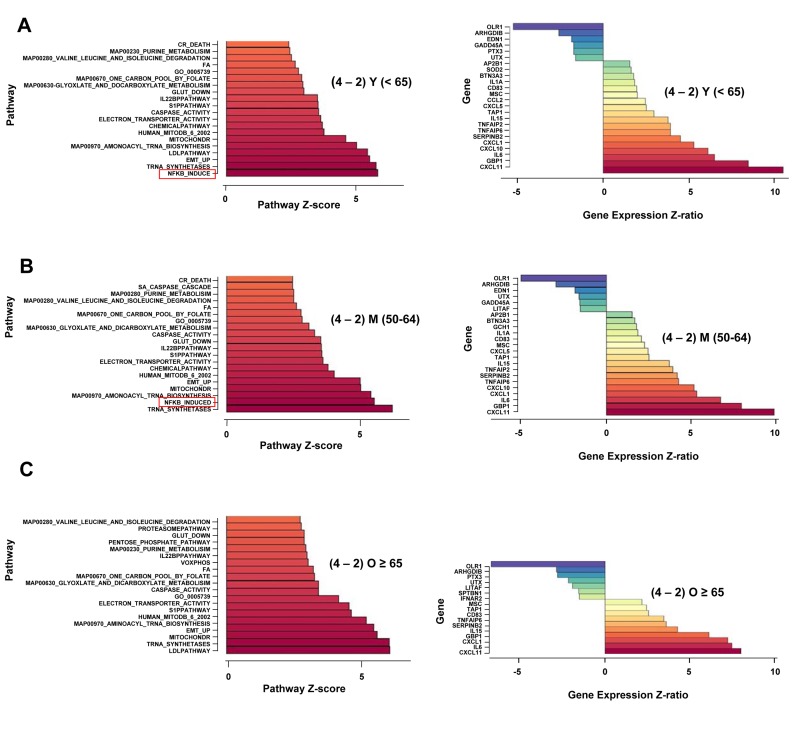
Age-associated alterations in NF-κB-dependent gene regulation in activated human peripheral blood CD4^+^ T lymphocytes Total RNA was prepared from CD4^+^ T cells incubated at 37°C for 4h without further treatment, or treated with plate-bound anti-CD3 for 2h and 4h. Fluorescently labeled cRNA was hybridized to Illumina HumanRef-8 Expression BeadChip as described in the Methods Section. A, B, C left panels. Normalized hybridization data was analyzed using Parametric Analysis of Gene Set Enrichment (PAGE) (Broad Institute, M.I.T., Cambridge MA). Pathway Z-scores were averaged amongst less than 65 (Y), 50-64 (M), 65 and older (O) subjects. Pathways that were up-regulated after 2h or 4h of activation compared to untreated cells were first determined (Figure S4A). Thereafter, the top 20 pathways that continued to be up-regulated between 2h and 4h of activation were identified (y axis). The Z-score difference between 2h and 4h of activation is indicated in the x axis. Right panels ‘NF-κB induced’ pathway genes that were altered between 2h and 4h of activation are listed (y axis); Z-ratios for the difference in expression between 2h and 4h are shown on the x axis. Positive numbers indicate genes that are up-regulated between 2h and 4h; negative numbers denote genes that are down-regulated between 2h and 4h. Z-ratio values were greater than 1.5, *p*-values for all genes were <0.05.

To examine the transcriptional response to anti-CD3 treatment, we analyzed RNA obtained from these cells using Illumina microarrays. PAGE analysis of the normalized data sets showed that the ‘NF-κB-induced’ pathway was the most prominent one induced after 2h or 4h anti-CD3 treatment regardless of age (Figure S4A), thereby validating the choice of activation times for functional analysis of NF-κB responses. Within the NF-κB induced pathway, cytokine and chemokine genes were amongst the most highly activated genes at both time points, and in both subject groups (Figure S4B, S4C). The pattern of nuclear RelA induction predicted that NF-κB-dependent gene expression would continue to increase between 2h and 4h of activation in the younger, but not older, participants. To test this, we evaluated pathway changes that occurred between 2h and 4h of activation. Though, 17 out of the top 20 pathways that increased between 2h and 4h of activation remained the same in the two age groups, we found that the ‘NF-κB induced’ pathway increased significantly only in the young group (Figure 2A) but not the old group (Figure 2C). Pathway analysis comparing 50-64 year old donors to the ≥ 65 group showed the same pattern (Figure 2B). We conclude that NF-κB transcriptional function is not sustained in activated CD4^+^ T cells from the elderly, thereby correlating closely with the biochemical assays of NF-κB induction.

To critically test the idea that NF-κB activation was not sustained in the elderly, we identified putative NF-κB target genes that were induced between 2h and 4h of activation. We found that in the younger and middle-age cohorts, 18 genes within this pathway were significantly up-regulated between 2h and 4h, whereas only 10 genes were significantly up-regulated in the older group (Figure 2A-C). Prominently missing from the older group were genes encoding CXCL10, TNFAIP2, CCL2, CXCL5, and IL-1α. Moreover, several genes that continued to be up-regulated in the older group had lower Z ratios than the same genes in the younger group. These genes included *CXCL11* (Z-ratio(O) = 8.02; Z-ratio (Y) = 10.54); *GBP1* (Z-ratio(O) = 6.14; Z-ratio(Y) = 8.51), and *SERPIN B2* (Z-ratio (O) = 3.6; Z-ratio (Y) = 4.51). Thus, more than half of “NF-κB-induced” genes that were up-regulated between 2h and 4h in the younger group were attenuated in the older group. Together, this set of genes identified the transcriptional consequences of the lack of continued NF-κB activation in response to TCR stimulation in CD4^+^T cells from older donors.

Interestingly, not all NF-κB target genes were reduced in expression between 2h and 4h of activation in the older cohort. Of particular interest, expression of *IL-6*, *CXCL1*, *CXCL11*, and *IL-15* continued to increase in CD4^+^ T cells from older individuals. Continued up-regulation of these genes in the absence of sustained NF-κB induction revealed regulatory differences between classes of NF-κB target genes. While expression of many target genes correlated well with NF-κB inducibility, others deviated considerably. This dichotomy was especially apparent in samples from older donors. We note that many of the discordantly regulated genes have been previously associated with aging. Serum IL-6 levels increase in the elderly [2, 3], yet several genome-wide analyses of aging tissues from humans and mice failed to uncover the source of systemic IL-6 [29-34]. Our observations suggest that inefficient down-regulation of IL-6 during T cell immune responses in the elderly may be one source of age-associated IL-6. Along with IL-6, CXCLl is one of the cytokines associated with senescence-associated secretory program (SASP) [35, 36]. It is intriguing that TCR activation of CD4^+^ T cells from older individuals induces some aspects of SASP. Finally, elevated IL-15 levels have been noted in age-associated osteoarthritis and very old individuals [37, 38].

**Figure 3:**
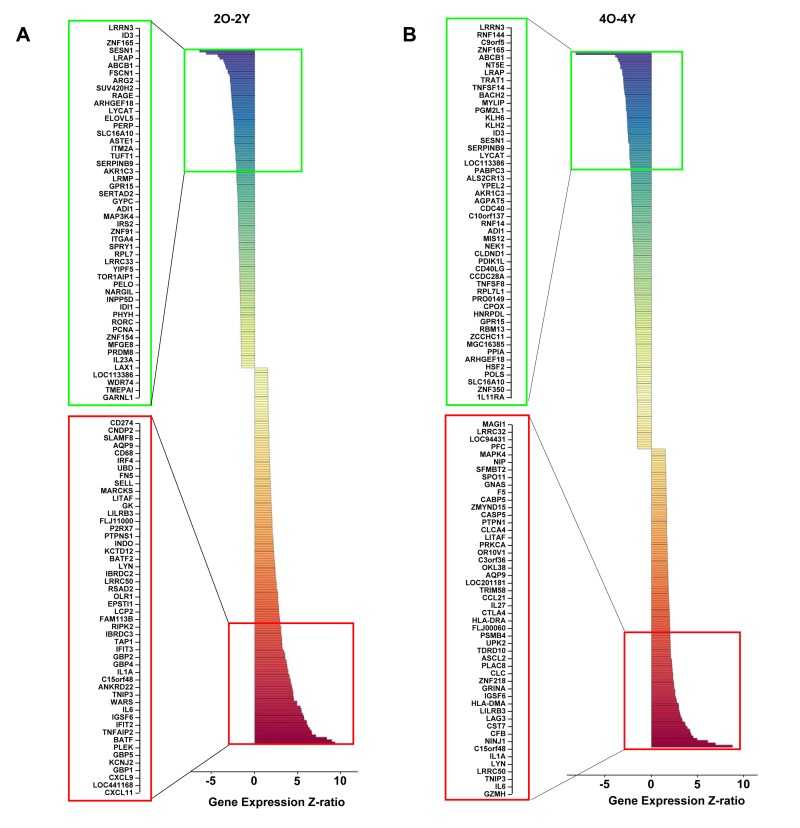
Age-associated alterations in TCR-inducible gene expression in human peripheral blood CD4^+^ T cells Total RNA prepared from anti-CD3 stimulated cells for the indicated times was converted to cRNA and hybridized to Illumina HumanRef-8 Expression BeadChip as described in Methods. The average expression level for each gene in the younger group (<65) was compared to the expression level for each gene in the older group (≥65). Genes whose average expression in Y and O groups differed by a Z-ratio >1.5, and *p*-value <0.05 were classified as being significantly different. The top 50 differentially up- (red) and down- (green) regulated genes after 2h (**A**) and 4h (**B**) of activation are shown. The total number of differentially up-regulated genes were 156 (2h) and 128 (4h). The total number of differentially down-regulated genes were 132 (2h) and 170 (4h).

To more broadly identify age-associated changes in response to TCR signaling we compared the pattern of inducible gene expression in cells from the Y and O age groups activated for 2h or 4h. At both activation time points, many genes were differentially expressed in the two groups; the top 50 genes up-or-down regulated in the ≥ 65 group compared to the ≤ 65 group are shown in Figure 3. Only a small proportion of these genes fell in the NF-κB induced category showing that many other pathways were also affected in cells from older donors. Putative NF-κB target genes that were consistently up-regulated in cells from older individuals included the cytokines IL-1α and IL-6. Additionally, up-regulation of *GZMH*, which is normally restricted to NK and CD8 cells, in cells from older individuals was interesting from the perspective that aged lymphocytes are known to express NK cell-specific cell surface receptors [39, 40]. Thus, older CD4 ^+^ T cells express several aspects of the NK cell gene program. The combination of NK receptors and GZMH may permit cytolytic activity of such cells.

**Figure 4:**
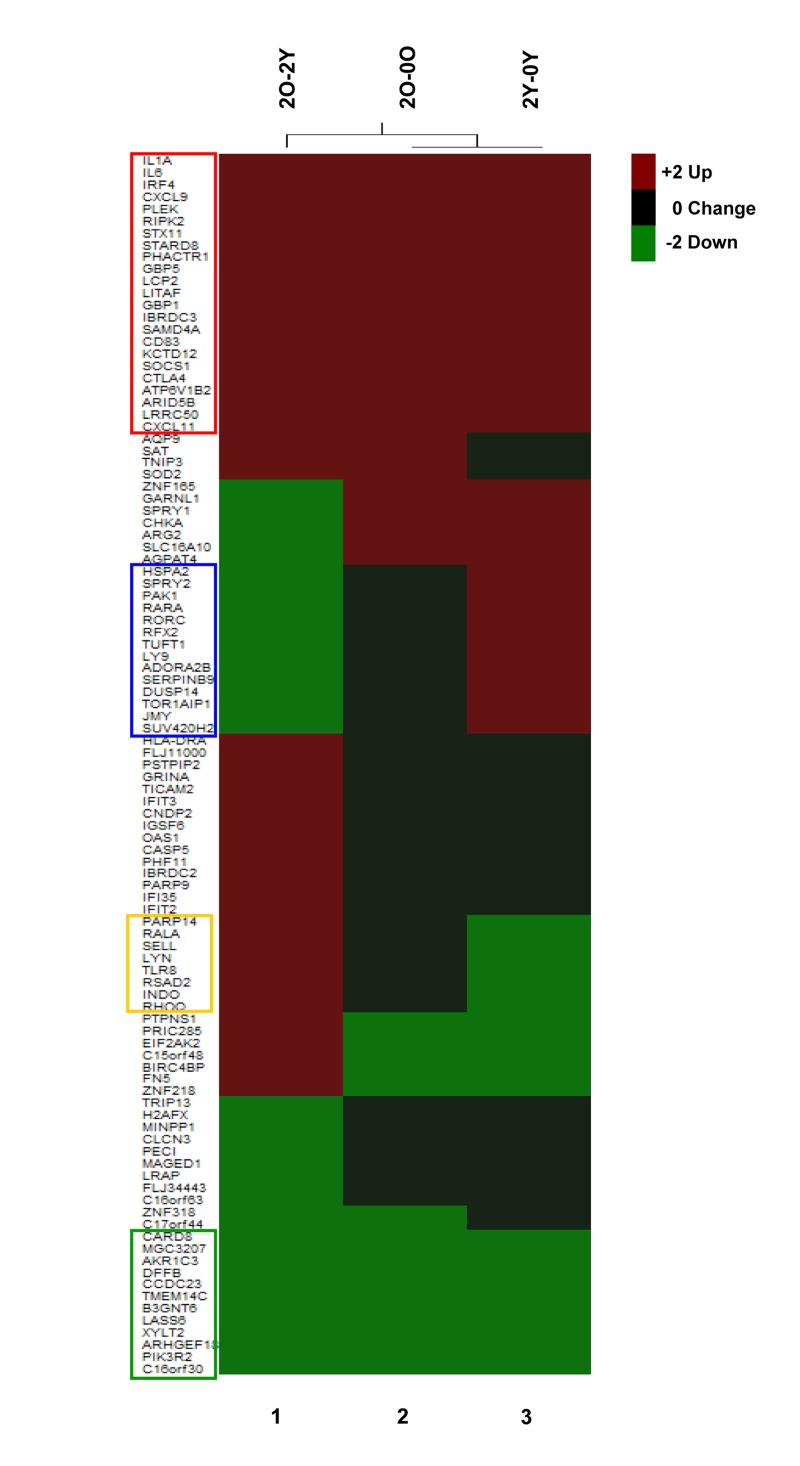
Age-associated changes in induction cha-racteristics of differentially expressed genes Top 50 differentially up- (red) and down- (green) regulated genes in CD4+ T cells from Old (O) (≥65) and Young (Y) (<65) donors after 2h TCR activation. These genes were selected based on the absolute value of the Z-ratios among the statistically significant selected genes (2O-2Y, column 1), O (column 2) and the Y (column 3). Genes highlighted within the red box were induced in both O and Y groups, the green box were repressed in both O and Y groups, the blue box genes were induced in Y but not in O groups, and the yellow box were repressed in Y not in the O group.

We further probed the nature of age-associated changes in TCR inducible gene expression. One perspective was provided by investigating the induction characteristics of genes whose expression was significantly different between Y and O cells after 2h activation (Figure 4, 2O-2Y). Amongst genes that were over expressed in the O group after 2h anti-CD3 treatment (Figure 4, 2O-2Y), we found 23 genes that were significantly induced in both Y and O groups (Figure 4, red box); our interpretation is that these genes are hyper-induced in the O group. Conversely, amongst genes whose expression was attenuated in the O group after 2h of anti-CD3 treatment, we found 12 genes that were repressed in both groups (Figure 4 green box); we infer that these genes are hyper-repressed in the O group. Additionally, we found genes that were induced in Y but not in O and thereby fell in the down-regulated category in a comparison of O and Y after 2h activation (Figure 4, blue box). Conversely, some genes that were turned down in response to TCR signaling in Y but not in O fell in the up-regulated category in the comparison of O and Y after 2h activation (Figure 4 yellow box). Thus, age-associated alterations in TCR signaling are reflected in complex transcriptional responses that affect inducible gene activation as well as repression. Similar categories could be seen after 4h anti-CD3 treatment (Figure S5).

## DISCUSSION

Our studies provide the first view of age-associated alteration of inducible gene regulation in response to TCR signaling in human CD4^+^ T cells. We used short-term anti-CD3 activation to focus on direct consequences of TCR signaling and, in particular, on the effects of age on NF-κB induction and NF-κB dependent gene expression. Using cells obtained from cytapheresis of subjects enrolled in the Baltimore Longitudinal Study on Aging, we show that NF-κB RelA nuclear translocation was induced similarly in CD4^+^ T cells from young and old subjects, but induction was not sustained in cells from older individuals. The biochemical data was reflected in reduced induction of putative NF-κB target gene transcription in the older cohort. However, mRNA levels of a subset of NF-κB target genes continued to increase in the elderly even after nuclear RelA levels reached a plateau. These observations indicate that age-associated NF-κB dysregulation differentially impacts different sets of NF-κB target genes. Importantly, we found that NF-κB target genes that were over-expressed in the older group included genes that have been implicated in age-associated chronic inflammation such as *IL-6*, *IL-1* and *IL-15*, and those involved in cellular senescence such as *CXCL1*. In the course of the biochemical studies, we also showed that standard normalization proteins, such as actin or the transcription factors SP1 and TBP, vary sufficiently between subjects that they cannot be used to compare between individuals.

Although our studies were conducted with a mixed population of CD4^+^ naïve and memory cells, we believe that the age-associated changes noted here do not reflect differences in responsiveness of naïve and memory cells. Firstly, the changes in putative NF-κB-dependent genes were apparent in a comparison between middle-aged and older donors where the population of naïve and memory cells are very similar. Secondly, the genes that we propose to be differentially regulated with aging were not identified as being differentially expressed in naïve and memory CD4^+^ T cells in earlier studies [41, 42]. We therefore propose that dysregulation of genes such as *IL-1*, *IL-6* and *IL-15* reflect true age-associated changes in CD4^+^ T cell responses. Whether these changes occur in one or both subsets of CD4^+^ T cell remains to be determined.

We propose that the differential effect on NF-κB target genes may, in part, explain the co-existence in older individuals of a background mild pro-inflammatory state and reduced ability to mount inflammatory immune responses. We suggest that those NF-κB target genes that require continued NF-κB activation contribute to effective immune response, for example, via activation of chemokines and their receptors. Reduced expression of such genes would result in impaired immunity in the elderly. At the same time, some NF-κB target genes such as *IL-6*, *IL-1*, and *IL-15* appear not to be exclusively dependent on NF-κB levels and, in some cases, are even hyper-induced in cells from older individuals. We suggest that this form of dysregulation may contribute over time to chronic inflammation seen in the elderly. An important corollary of the hypothesis is that understanding the regulatory differences between the two kinds of NF-κB-dependent gene transcription may provide insights into the origins of age-associated chronic inflammation. Additionally, our observations implicate CD4^+^ T cells as one source of age-associated inflammatory cytokines.

The majority of genes that were differentially expressed in activated cells from older individuals were not classifiable as NF-κB target genes. Clearly, inducible gene expression mediated by transcription factors other than NF-κB is also affected by age. The altered patterns of gene expression arose in different ways. For example, genes that were up-regulated in O compared to Y after 2h activation included genes that were induced to higher levels in O than in Y (hyper-induced) or genes that were inducibly suppressed in Y but not in O. Conversely, genes that were down- regulated in O compared to Y after 2h activation included genes that were repressed more in O than in Y (hyperrepressed) or genes that were activated in Y but not in O. The aggregate of these different kinds of changes results in widespread alterations of gene expression in TCR-activated CD4^+^ T cells from older individuals. It will be important to identify which changes are most important in contributing to the overall attenuation of CD4^+^ T cell responses in the elderly.

Finally, it is interesting to compare our results in human CD4^+^ T cells with those of earlier studies that examined age-associated inducible gene expression in murine T cells, particularly with regard to inflammation-related genes. Despite the use of different T cell populations, up-regulation of the chemokines CCL3 and CCL4 and the cytokine IFN-γ was noted in several of the murine studies [23, 24, 26]. Additionally, up-regulation of the cytokines IL-4, IL-10, and IL-17 was noted in a different subset of mouse studies [20, 26]. However, these genes did not score as being differentially expressed in human CD4^+^ T cells from older individuals at least at the activation time points used here. Instead, we found *IL-1 α*, *IL-6* to be prominently up-regulated in human CD4^+^ T cells from older individuals. A common feature between the mouse and human studies was the differential up-regulation of *CXCL9* and *CXCL11* in the older cohorts. These differences highlight the importance of continued analysis of human samples for the study of aging.

## METHODS

### Subjects

31 donors (Table S1) of age range 25-81 years (17 male and 14 female) were enrolled in this study. All blood samples were taken after permission from the individuals in the study. The subjects for this study were selected from the Baltimore Longitudinal Study on Aging (BLSA). BLSA participants are volunteers that at the time of study enrollment are “healthy” based on very strict eligibility criteria, which include no major chronic diseases (such as coronary heart disease, congestive heart failure, peripheral artery disease, cancer, chronic pulmonary diseases, diabetes, severe osteoarthritis and others), no chronic drug treatment except low dose Aspirin, antihypertensive drugs and statins, no mobility impairment and or disability (ability to walk 400 meters without stopping and without developing symptoms), no cognitive impairment (Mini Mental State Examination score >26 and less that 3 errors in the Blessed Mental Status), no joint replacement, no osteoporotic fractures. Thus, BLSA participants tend to be healthier than the general population. In addition, this study required a large quantity of WBC that could only be obtained by cytapheresis. Eligibility for cytapheresis in the BLSA is based on even more strict criteria based on the American Association for Blood Bank Criteria for whole blood donation. Thus, participants in this study were exceptionally healthy, and therefore, the difference between the two groups should be attributable to aging “per se” BLSA has continuing approval from the Institutional Review Board of the MedStar Research Institute. Participants provided informed consent for all analyses included in this study.

### Cell preparation

Peripheral blood mononuclear cells (PBMC) were isolated from heparinized blood using Ficoll-Paque Plus (GE Healthcare) density gradient centrifugation. Cells were washed 3-4 times in phosphate buffered saline (PBS) at room temperature prior to purification of CD4^+^ T cells by positive selection using anti-human CD4 microbeads (Miltenyi Biotec). The purity of CD4^+^ T cells used in this study typically ranged from 80-94 % as determined by flow cytometry after gating out debris and dead cells (Figure S1 and summarized in Table S1). Two samples out of the 31 had lower CD4^+^ purity (64% and 76%). Cell viability as determined by trypan blue dye exclusion was greater than 90%.

### CD4^+^ T cell activation

Six well plates were incubated with rabbit anti-mouse IgG (20μg/ml) (Zymed Laboratories Inc.) overnight at 4°C. Plates were rinsed with cold PBS twice and once with RPMI 1640 medium followed by adding of anti-CD3 mAb (2μg/ml) (BD PharMingen) incubated 2h at 37°C. 6×10^7^ CD4^+^ T cells in 18 ml culture medium were added to Ab-coated plates. Control cells were plated in dishes that did not contain anti-CD3 mAb. Cells were incubated for 0, 2, 4 h in 5% CO_2_ at 37° C. Cells were collected by centrifugation and used to prepare nuclear (NE) and cytoplasmic extracts (CE) as described previously [43, 44].

### Western blotting

Extracts were fractionated by electrophoresis through 10% SDS-poly-acrylamide gels, transferred to Immuno-Blot PVDF membranes (Bio-Rad) and probed with primary antibody [NF-κB p65 (C-20) rAB at 1:1000 (Santa Cruz), Sp1 (H-225) rAB at 1:1000 (Santa Cruz), TBP mAB at 1:500 (Abcam), beta actin mAb at 1:1000 (Abcam)]. Antibody-bound proteins were detected after treatment of membrane with horseradish peroxidase-conjugated goat anti-rabbit IgG, or rabbit anti-mouse IgG followed by visualization using enhanced chemiluminescence (ECL Pierce). Quantitation analysis was performed with Image Quant computer program (GE Healthcare).

### Quantification of p65/RelA levels

Control proteins (SP1, β-actin and TBP) were used to compare between extracts from the same individual (Fig. S2A). Levels of all three proteins varied sufficiently between individuals (Fig. S2B) to preclude any meaningful inter-individual comparisons. Equal amounts of nuclear extracts were used and the level of RelA compared to a serially diluted control extract (Fig. S2C and Table S2). After autoradiography films scanned using Epson Perfection 4990 Photo scanner and the image imported into Image Quant TL (GE Healthcare). Quantitation of p65/RelA and control proteins was carried out in accordance with the manufacturer's instruction for quantity calibration. A standard curve based on the intensity of control cytosolic extracts was plotted, from which absolute levels of p65/RelA in test nuclear extracts were calculated for each sample (expressed in arbitrary units). Data are presented in Table S3 and S4. For statistical analysis, Student's t-tests (paired or unpaired) were used (*p*<0.05 was considered as significant).

### Total RNA purification

Total RNA was extracted from frozen cell pellets (5×10^6^cells) using the Qiagen RNeasy Mini Kit (QIAGEN Inc, Valencia, CA, USA). Prior to microarray analysis the RNA quality and quantity were checked using an Agilent 2100 bio-analyzer and RNA nano chips.

### Total RNA purification and microarray hybridizations

Total RNA extracted from frozen cell pellets (5×10^6^ cells) using the Qiagen RNeasy Mini Kit (QIAGEN Inc) was used to generate biotin-labeled cRNA using the Illumina TotalPrep RNA Amplification Kit (Ambion) according to the manufacturer's recommendation. A total of 0.75μg of biotin-labeled cRNA was hybridized at 58°C for 16 hours to Illumina's Sentrix HumanRef-8 Expression BeadChips (Illumina). The arrays were washed, blocked and the labeled cRNA was detected by staining with streptavidin-Cy3. The arrays were scanned using an Illumina BeadStation 500X Genetic Analysis Systems scanner and the image data extracted using Illumina BeadStudio software, version 3.0.

### Microarray data analysis

Microarray data was analyzed using DIANE 6.0, a spreadsheet-based microarray analysis program based on SAS JMP7.0 system [45]. Raw microarray data were subjected to filtering by the detection *p*-values and than normalized by Z-transform with log signal values; the data are further tested for significant changes as previously described [46]. The sample quality was first analyzed by scatter plots, principal component analysis, and gene sample Z-score based hierarchy clustering to exclude possible outliners. One-way independent samples ANOVA test was used to eliminate the genes with larger variances within each comparison group with *p* value cutoff 0.05. Individual genes with t-test *p*-value ≤ 0.05, absolute value of Z-ratio ≥ 1.5 and fdr ≤ 0.3 and with no negative average Z-score across samples were considered significantly changed. Hierarchical clustering/K-means clustering and Principal Components Analysis (PCA) was performed to identify clustering within groups. Array data for each experimental donor was also originally hierarchically clustered in Illumina Bead Studio version 2.0. All of the results were presented graphically as well as by spreadsheets.

The Parametric Analysis of Gene Enrichment (PAGE) algorithm [47] was employed for gene set enrichment analysis by using all of the genes in each sample as input against and the data set supplied by Gene Ontology Institute and pathway gene set of MIT Broad Institute molecular signature database [48]. For each relevant comparison, the lists of differentially expressed genes and Z-ratios were entered into the PAGE Pathway Analysis software to organize them according to known biological pathways. The enrichment Z-scores for each functional grouping were calculated based on the chance of mRNA abundance changes predicting these interactions and networks by Z-test. The *p*-value was calculated by comparing the number of user-specified genes of interest participating in a given function or pathway relative to the total number of occurrences of these genes in all pathway annotations stored in the knowledge base. All of the pathways must at least have three genes found in the microarray gene set. Pathways *p*-value ≤ 0.05 and pathways fdr ≤ 0.3 are the cutoff criteria for the significant pathway selection. The significant pathway results are further presented by cluster heat map for their association relations and by bar plot for their change patterns. The related gene sets in each pathway plus their expression patterns and the related pathway for each gene are also supplied. The genes in the pathway groups are supplied and their expression patterns are also indicated. Each gene's pathway information is also available.

## SUPPLEMENTAL DATA


