# Supplementary material for: Age-associated alterations in inducible gene transcription in human CD4+ T lymphocytes
Source: Aging (Albany NY). 2013 Jan 30;5(1):18–36. doi: 10.18632/aging.100522 (PMC3616229; doi:10.18632/aging.100522)
Supplement: Supplementary file 1 [file aging-05-018-s001.pdf]

modifications in circulating IL-15 levels in humans. Mediators Inflamm. 2005; 2005:245-247.

39. van Bergen J, Thompson A, van der Slik A, Ottenhoff TH, Gussekloo J and Koning F. Phenotypic and functional characterization of CD4 T cells expressing killer Ig-like receptors. J Immunol. 2004; 173:6719-6726.

40. van Bergen J, Kooy-Winkelaar EM, van Dongen H, van Gaalen FA, Thompson A, Huizinga TW, Feltkamp MC, Toes RE and Koning F. Functional killer Ig-like receptors on human memory CD4+ T cells specific for cytomegalovirus. J Immunol. 2009; 182:4175-4182.

41. Liu K, Li Y, Prabhu V, Young L, Becker KG, Munson PJ and Weng N. Augmentation in expression of activation-induced genes differentiates memory from naive CD4+ T cells and is a molecular mechanism for enhanced cellular response of memory CD4+ T cells. J Immunol. 2001; 166:7335-7344.

42. Hess K, Yang Y, Golech S, Sharov A, Becker KG and Weng NP. Kinetic assessment of general gene expression changes during human naive CD4+ T cell activation. Int Immunol. 2004; 16:1711-1721.

43. Tam WF, Lee LH, Davis L and Sen R. Cytoplasmic sequestration of rel proteins by IkappaBalpha requires CRM1-dependent nuclear export. Mol Cell Biol. 2000; 20(6):2269-2284.

44. Banerjee D, Liou HC and Sen R. c-Rel-dependent priming of naive T cells by inflammatory cytokines. Immunity. 2005; 23:445-458.

45. Garg S, Nichols JR, Esen N, Liu S, Phulwani NK, Syed MM, Wood WH, Zhang Y, Becker KG, Aldrich A and Kielian T. MyD88 expression by CNS-resident cells is pivotal for eliciting protective immunity in brain abscesses. ASN Neuro. 2009; 1(2).

46. Cheadle C, Vawter MP, Freed WJ and Becker KG. Analysis of microarray data using Z score transformation. J Mol Diagn. 2003; 5:73-81.

47. Kim SY and Volsky DJ. PAGE: parametric analysis of gene set enrichment. BMC Bioinformatics. 2005; 6:144.

48. Subramanian A, Tamayo P, Mootha VK, Mukherjee S, Ebert BL, Gillette MA, Paulovich A, Pomeroy SL, Golub TR, Lander ES and Mesirov JP. Gene set enrichment analysis: a knowledge-based approach for interpreting genome-wide expression profiles. Proc Natl Acad Sci U S A. 2005; 102:15545-15550.

SUPPLEMENTAL DATA

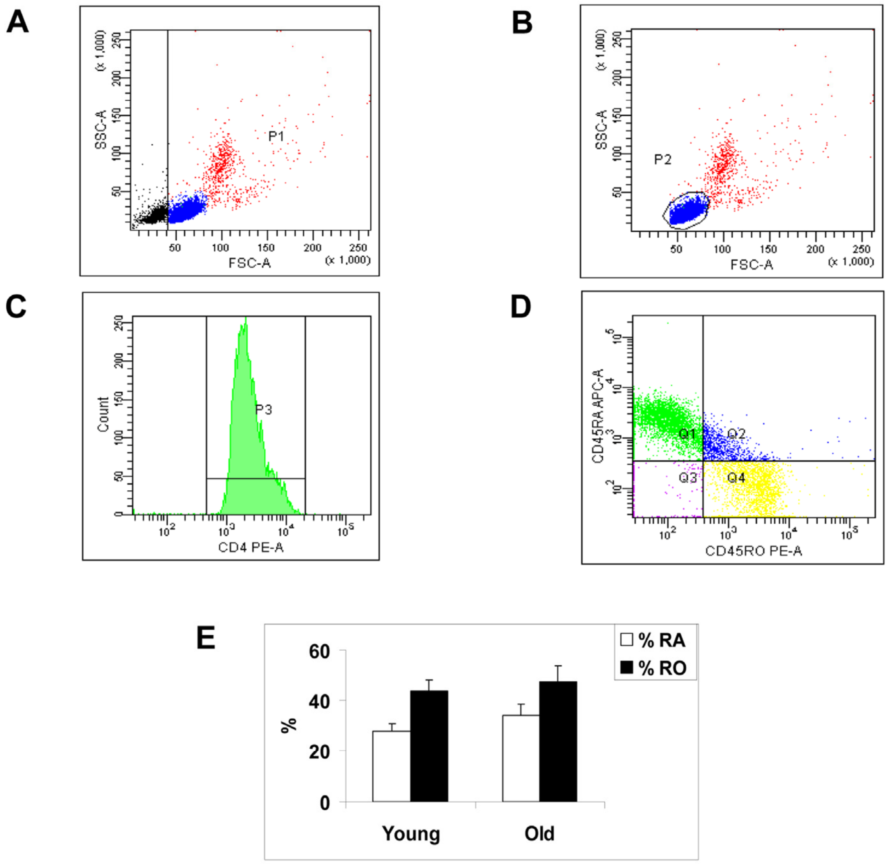

Figure S1. Flow cytometric analysis of cell populations used in this study. Representative example of flow cytometric analysis from one donor to indicate gates used in this study. Anti-CD4-PE mAb, anti-CD45RA-APC mAb, anti-CD45ROPE mAb were used for flow cytometry. Dead cell and debris were excluded using P1 gate (**A**); the percentage of lymphocytes within P1 was quantitated using P2 gate (**B**). The majority of cells in P2 were CD4+ T cells (**C**). Distribution of CD45RA+ and CD45RO+ cells within P2 was determined by dual staining with appropriate antibodies (**D**). Percentage of cells in quadrants Q1 and Q4 were denoted CD45RA+ and CD45RO+, respectively (summarized in Table S1). (**E**) Average proportions of CD45RA+ and CD45RO+ cells in donors less than 65 years of age (Young) or 65 years and higher (Old) obtain from the data in Table S1. The comparable proportions of CD45RA+ and CD45RO+ cells in our Y and O cohorts is probably because RA+/RO+ ratio levels off in CD4+ T cells between second and third decile of life (Cossarizza *et al.* 1996 Mech. Ageing Dev. 86:173-95). In our Y cohort the number of 20-39 year-olds is 4 out of a total of 20.

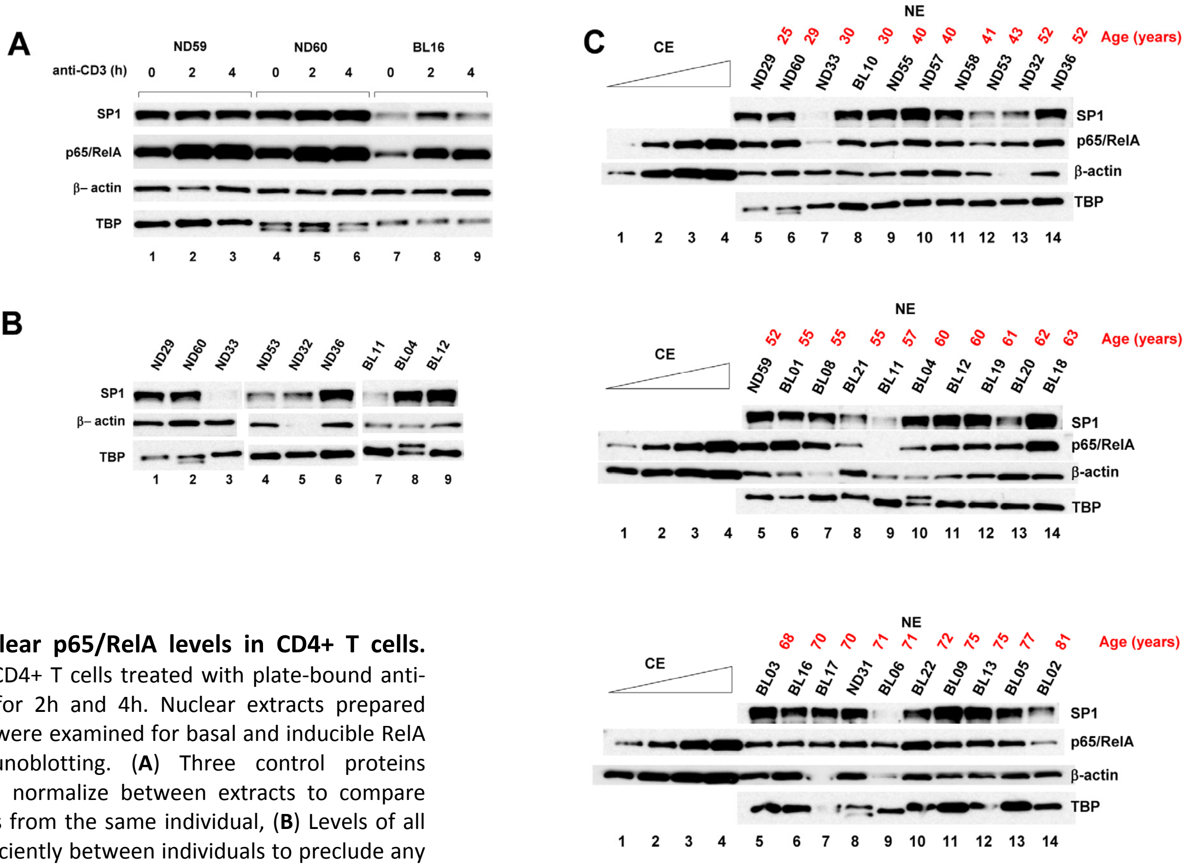

Figure S2. Nuclear p65/RelA levels in CD4+ T cells. Primary human CD4+ T cells treated with plate-bound anti-CD3 antibodies for 2h and 4h. Nuclear extracts prepared from these cells were examined for basal and inducible RelA levels by immunoblotting. (**A**) Three control proteins typically used to normalize between extracts to compare between extracts from the same individual, (**B**) Levels of all three varied sufficiently between individuals to preclude any meaningful interindividual comparisons. (**C**) Nuclear p65/RelA expression in unactivated human peripheral blood CD4+ T lymphocyte. Purified CD4+ T cells were incubated at 37oC for 4h in the absence of anti-CD3 antibody. Nuclear extracts (NE) were fractionated by SDS-PAGE, transferred to PVDF membranes and probed with antibodies directed against p65/RelA, transcription factors SP1 and TATA binding protein (TBP), or β actin. Each gel has nuclear extracts from 10 donors (indicated above the lanes) arranged by age. Equal amounts of nuclear extracts (NE) from untreated cells used and compared basal p65/RelA levels between individuals to a serially diluted control cytoplasmic extract (CE) shown in lanes 1-4 of each gel.

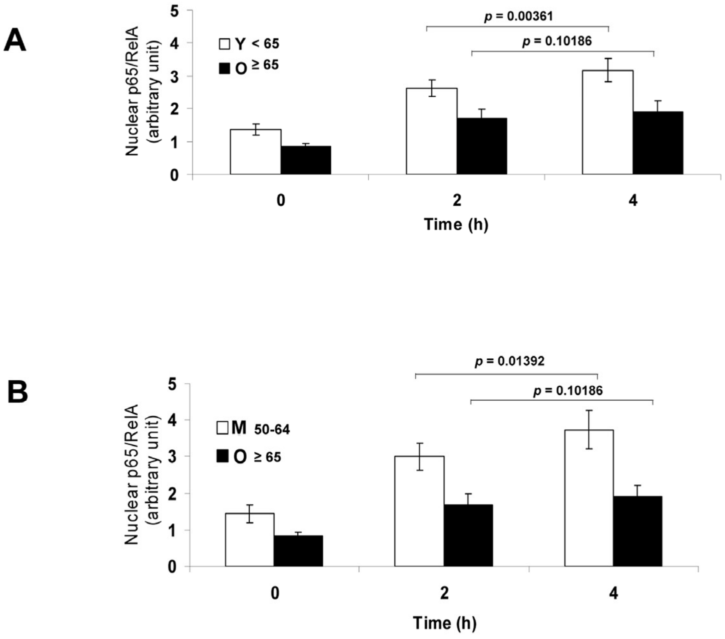

Figure S3. Anti-CD3-activated CD4+ T cells. (**A**) Measure of absolute p65/RelA levels in anti-CD3-activated CD4+ T cells. p65/RelA levels from columns 7-9 in Table S4 were averaged into two groups including subjects younger than 65 (Y), or 65 years and older (O), and graphed. Error bars reflect the standard error of the mean (±SEM) (in young 0.16; 0.26; 0.36) (in old 0.08; 0.30; 0.33) (**B**) Same analyze done for subjects middle age (M) (50-64) and 65 years and older. Error bars reflect the standard error of the mean (±SEM) (in middle 0.24; 0.37; 0.52) (in old 0.08; 0.30; 0.33)

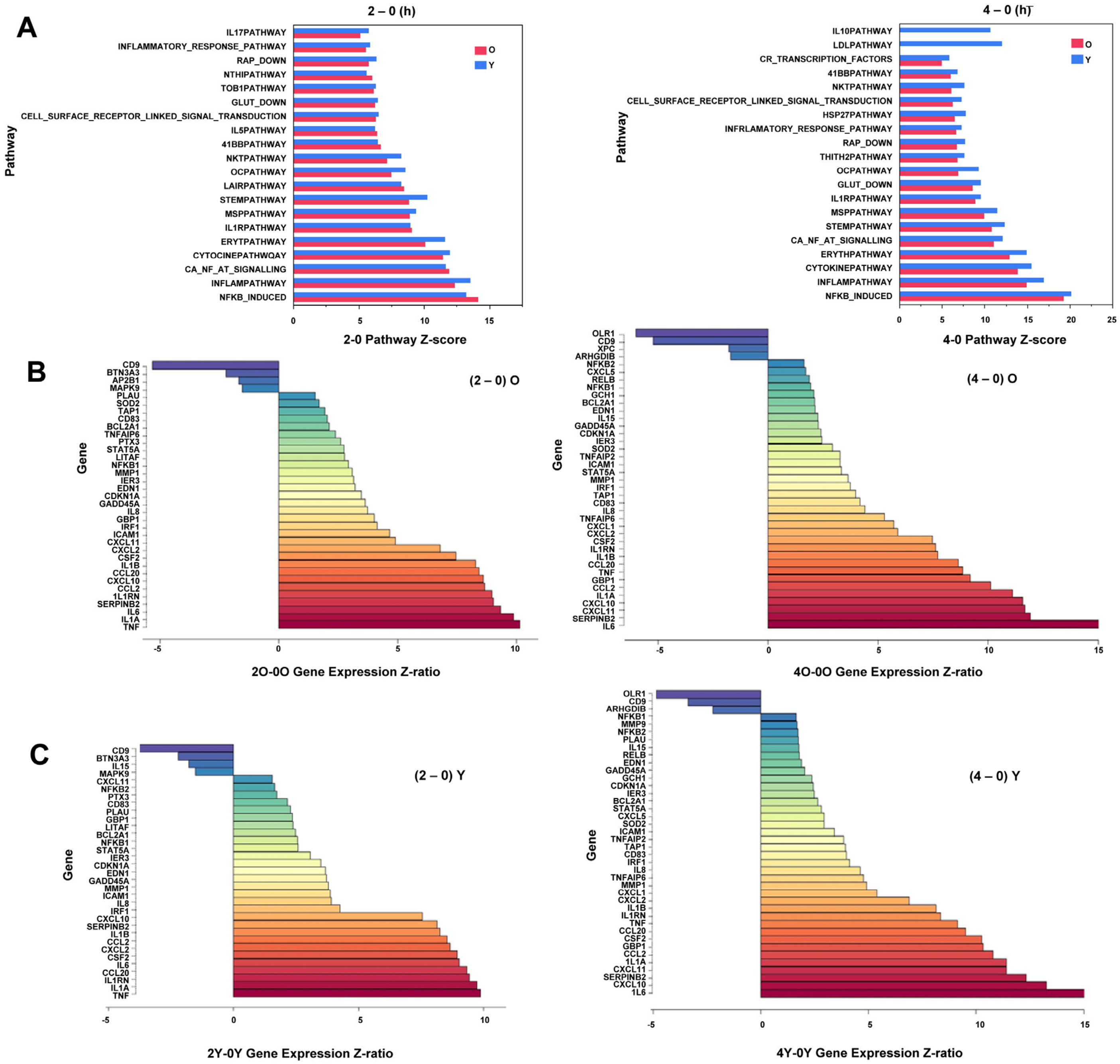

Figure S4. NF-κB induce pathway gene expression in anti-CD3 activated human peripheral blood CD4+ T lymphocytes. (**A**) Normalized hybridization data was analyzed using PAGE (Broad Institute, M.I.T., Cambridge MA). Top 20 Pathway Z-scores after 2h (left panel) and 4h (right panel) of activation were averaged between less than 65 (Y) and 65 and older (O) subjects and the Z-score difference between O and Y is shown on the X-axis. Blue and red bars represent younger and older subjects, respectively. Each row denotes a different pathway. Z-score differences shown were statistically significant by pathways *p*-value ≤ 0.05 and pathways fdr ≤ 0.3, (**B**) Significantly induced genes in the NF-κB -induced pathway after 2h (left) or after 4h (right) activation in the older subject group and (**C**) in the younger subject group.

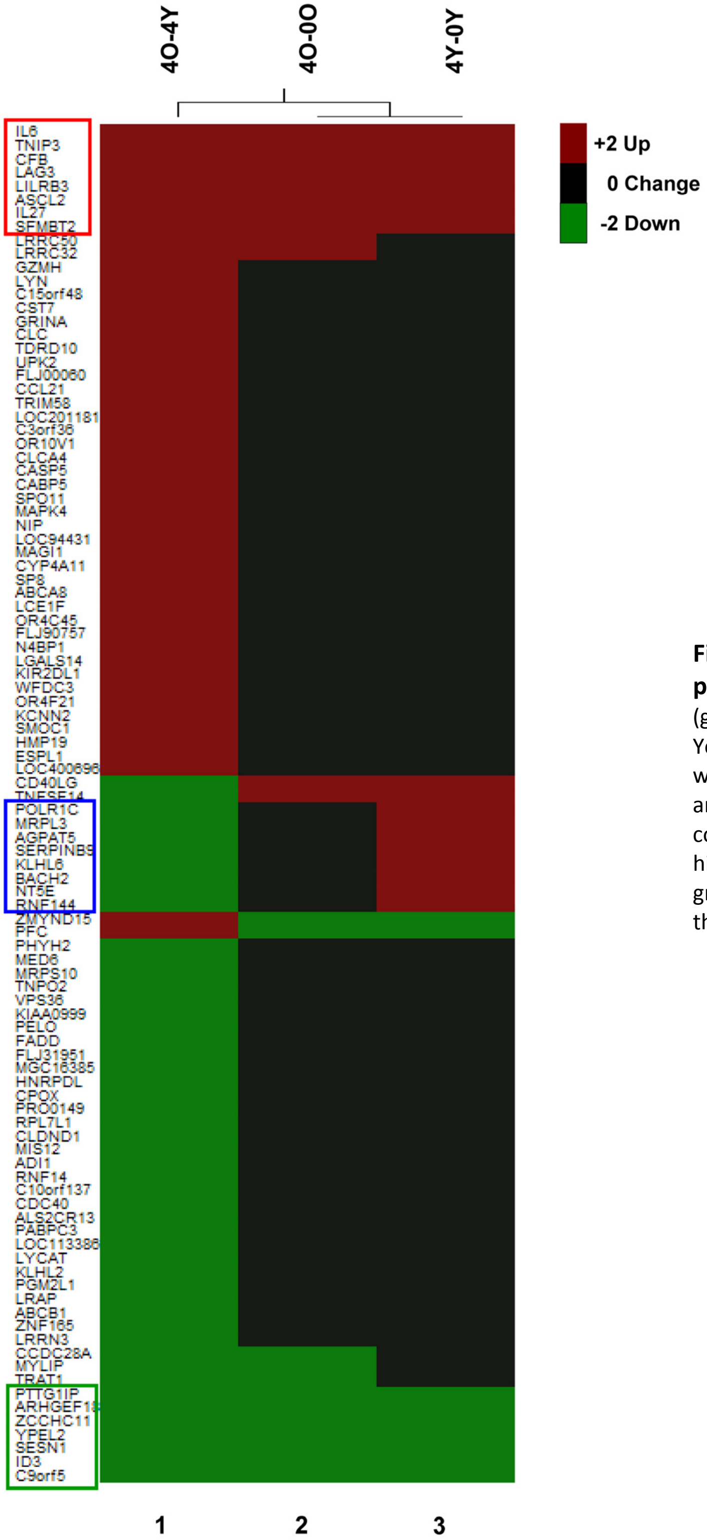

Figure S5. Age associated gene expression change pattern heat map. Top 50 differentially up- (red) and down- (green) regulated genes in CD4+ T cells from Old (O) (≥65) and Young (Y) (<65) donors after 4h TCR activation. These genes were selected based on the absolute value of the Zratios among the statistically significant selected genes (4O-4Y, column 1), O (column 2) and the Y (column 3). Genes highlighted within the red box were induced in both O and Y groups, the green box were repressed in both O and Y groups, the blue box genes were induced in Y but not in O groups.

Donor #	Sex	Age	lymphocytes % of P1	CD4+ % of P2	RA+ % of P2	RO+ % of P2	
ND29	M	25	92.3	99.6	49.2	41.1	<65
ND60	F	29	94.1	99.4	30.3	51.1	
ND33	M	30	87.2	99.0	21.8	73.1	
BL10	M	30	91.3	99.3	43.4	12.2	
ND55	M	40	92.1	99.0	29.3	32.7	
ND57	F	40	92.4	99.2	27.5	61.4	
ND58	F	41	87.7	98.7	27.2	44.6	
ND53	M	43	91.5	98.9	21.8	36.7	
ND32	M	52	86.9	99.0	28.5	12.2	
ND36	M	52	79.9	99.3	19.8	56.6	
ND59	M	52	94.0	99.5	15.0	38.4	
BL01	F	55	91.4	99.4	none	none	
BL08	F	55	91.3	99.4	28.6	60.2	
BL21	M	55	84.0	99.2	23.1	51.9	
BL11	F	57	89.6	99.2	27.5	32.5	
BL04	M	60	88.5	99.2	28.3	57.1	
BL12	M	60	86.0	98.4	12.4	42.9	
BL19	F	61	89.0	99.4	61.6	15.7	
BL20	F	62	64.3	98.1	5.5	85.1	
BL18	M	63	92.9	99.2	30.2	22.6	
BL03	F	68	83.4	98.7	41.9	45.2	≥65
BL16	F	70	76.4	98.6	65.1	3.7	
BL17	M	70	87.3	99.4	46.6	31.0	
ND31	F	71	88.6	99.5	23.8	68.3	
BL06	F	71	91.4	99.6	29.5	51.9	
BL22	M	72	88.6	99.5	20.2	63.1	
BL07	M	73	86.8	99.2	none	none	
BL09	M	75	93.8	99.5	40.2	34.6	
BL13	F	75	90.0	98.9	26.7	45.6	
BL05	M	77	89.2	99.4	26.1	58.9	
BL02	F	81	88.5	99.6	19.4	70.6	

Table S1. Summary of CD4+ T cell purity (column 5) and CD45RA+ and CD45RO+ distribution (columns 6 and 7) as determined by flow cytometry described in Fig. S1. ND indicates Normal Donor; BL indicates BLSA Donor. Younger (< 65 years) and older (≥ 65 years) cohort groups are indicated to the right of the table. CD4+ T cells were isolated from a total of 31 donors for this study whose donor number, sex and age are shown. BLSA marked donors were volunteers in the Baltimore Longitudinal Study on Aging.

A

	Controls	Age	Calib Vol(μg)
Dilution Controls	C		0.5
	C		1
	C		2
	C		4
Samples	ND29	25	1.43
	ND60	29	1.95
	ND33	30	0.5
	BL10	30	1.34
	ND55	40	1.09
	ND57	40	1.6
	ND58	41	1.21
	ND53	43	0.73
	ND32	52	1.03
	ND36	52	1.88

B

	Controls	Age	Calib Vol(μg)
Dilution Controls	C		0.5
	C		1
	C		2
	C		4
Samples	ND59	52	1.99
	BL01	55	2.7
	BL08	55	1.39
	BL21	55	0.58
	BL11	57	0.39
	BL04	60	0.68
	BL12	60	1.22
	BL19	61	1.18
	BL20	62	1.16
	BL18	63	3.16

C

	Controls	Age	Calib Vol(μg)
Dilution Controls	C		0.5
	C		1
	C		2
	C		4
Samples	BL03	68	0.95
	BL16	70	0.78
	BL17	70	0.78
	ND31	71	1.03
	BL06	71	0.73
	BL22	72	2.13
	BL09	75	1.11
	BL13	75	1.08
	BL05	77	0.86
	BL02	81	0.37

Table S2. Quantitation of p65/RelA expression in unactivated CD4+ T cells shown in Figure S2 C. Nuclear p65/RelA expression in 29 samples displayed in Fig S2 C. Sample BL22 was excluded from this analysis because the extract was derived from different cell numbers. After autoradiography the film was scanned using Epson Perfection 4990 Photo scanner and the image imported into Image Quant TL (GE Healthcare). Quantitation of p65/RelA and control proteins was carried out in accordance with the manufacturers instruction for Quantity calibration. A standard curve based on the intensity of control cytosolic extracts was plotted, from which absolute levels of p65/RelA in test nuclear extracts was calculated for each sample (expressed in arbitrary units).

Donors	(intensity of 0h)<br>0h	(intensity of 2h)<br>2h	(intensity of 4h)<br>4h
**ND29**	**201151.67**	**265305.75**	**328314.54**
**ND60**	**229366**	**372300**	**350679**
**ND33**	**106958.55**	**304729.97**	**442138.92**
**BL10**	**175860**	**380260**	**480010**
**ND55**	**326817**	**536422**	**550345**
**ND57**	**302072**	**417930**	**415452**
**ND58**	**234931**	**376926**	**429728**
**ND53**	**149734**	**272060.45**	**290108.5**
**ND32**	**67320**	**176096.5**	**240088.59**
**ND36**	**106056**	**226633.78**	**384849.92**
**ND59**	**197792**	**373070**	**379530**
**BL01**	**174162**	**322405.8**	**391507.4**
**BL08**	**178673**	**271626.18**	**418993.03**
**BL21**	**97497**	**231346**	**336739**
**BL11**	**21905.68**	**211165**	**259016**
**BL04**	**288381**	**356925**	**399146**
**BL12**	**201246**	**296932.04**	**206175.5**
**BL19**	**152084**	**317213**	**367644.63**
**BL20**	**66461**	**224702.5**	**301213**
**BL18**	**456552**	**612264**	**604314.5**
**BL03**	**131915**	**298762**	**319795.87**
**BL16**	**43834.79**	**212792**	**219384.5**
**BL17**	**241776**	**441338**	**533976.5**
**ND31**	**337006**	**404735**	**473846**
**BL06**	**254387.09**	**529930.65**	**634780.95**
**BL09**	**150339**	**219316**	**366784.56**
**BL13**	**184118**	**306192**	**306865.5**
**BL05**	**403559.5**	**482018**	**446672**
**BL02**	**219415**	**336449**	**270286**

Table S3. p65/RelA nuclear expression in human peripheral blood CD4+ T lymphocytes activated via the T cell antigen receptor. Quantitation of nuclear p65/RelA expression in anti-CD3 activated human CD4+ T lymphocytes. Nuclear extracts from untreated cells or cells treated for 2h and 4h were separated by SDS-PAGE followed by immunoblotting with anti-p65 antibodies described in Methods. Autoradiographs were scanned and quantitated using Image Quant TL software as described in the legend to Table S2. Numbers represent p65/RelA signal intensity in untreated cells (0h) and cells activated for 2h or 4h. Donor identification is provided in the left column.

Donors	Age	Y (<65) O (≥65)	(Normalize to 0h) 0h	(Normalize to 0h) 2h	(Normalize to 0h) 4h	(Calibrated 0h) 0h	(Calibrated 0h x Normalized 2h) 2h	(Calibrated 0h x Normalized 4h) 4h
ND29	25	Y	1	1.32	1.63	1.43	1.89	2.33
ND60	29	Y	1	1.62	1.53	1.95	3.17	2.98
ND33	30	Y	1	2.85	4.13	0.50	1.42	2.07
BL10	30	Y	1	2.16	2.73	1.34	2.90	3.66
ND55	40	Y	1	1.64	1.68	1.09	1.79	1.84
ND57	40	Y	1	1.38	1.38	1.60	2.21	2.20
ND58	41	Y	1	1.60	1.83	1.21	1.94	2.21
ND53	43	Y	1	1.82	1.94	0.73	1.33	1.41
ND32	52	Y	1	2.62	3.57	1.03	2.69	3.67
ND36	52	Y	1	2.14	3.63	1.88	4.02	6.82
ND59	52	Y	1	1.89	1.92	1.99	3.75	3.82
BL01	55	Y	1	1.85	2.25	2.70	5.00	6.07
BL08	55	Y	1	1.52	2.35	1.39	2.11	3.26
BL21	55	Y	1	2.37	3.45	0.58	1.38	2.00
BL11	57	Y	1	9.64	11.82	0.39	3.76	4.61
BL04	60	Y	1	1.24	1.38	0.68	0.84	0.94
BL12	60	Y	1	1.48	1.02	1.22	1.80	1.25
BL19	61	Y	1	2.09	2.42	1.18	2.46	2.85
BL20	62	Y	1	3.38	4.53	1.16	3.92	5.26
BL18	63	Y	1	1.34	1.32	3.16	4.24	4.18
BL03	68	O	1	2.26	2.42	0.95	2.15	2.30
BL16	70	O	1	4.85	5.00	0.78	3.79	3.90
BL17	70	O	1	1.83	2.21	0.78	1.42	1.72
ND31	71	O	1	1.20	1.41	1.03	1.24	1.45
BL06	71	O	1	2.08	2.50	0.73	1.52	1.82
BL09	75	O	1	1.46	2.44	1.11	1.62	2.71
BL13	75	O	1	1.66	1.67	1.08	1.80	1.80
BL05	77	O	1	1.19	1.11	0.86	1.03	0.95
BL02	81	O	1	1.53	1.23	0.37	0.57	0.46

Table S4. Nuclear p65/RelA expression levels in untreated (0h) cells, and after 2h and 4h of activation with anti-CD3. p65/RelA levels in CE titration are assigned the values 0.5, 1, 2, 4 in arbitrary units. Fold activation and absolute p65/RelA levels in human CD4+ T cells activated by anti-CD3 treatment. The fold activation in response to anti-CD3 was obtained by determining the increase in p65/RelA levels compared to that in untreated cells. For this, the intensity measurements for all samples from a subject (Table S3) were divided by the value in untreated cells. Thus, untreated cells from all subjects get a value 1 (column 4); columns 5 and 6 show the increase in p65/RelA after 2h or 4h anti-CD3 treatment compared to untreated cells. To obtain a measure of the absolute levels of p65/RelA in activated cells (columns 8 and 9) we multiplied the fold activation at each time point with the absolute level (in arbitrary unites) of p65/RelA in untreated cells obtained from the data in Fig. S2 C and Table S2, which is reproduced here in column 7.
